# Weight bias in mental health settings: a scoping review

**DOI:** 10.3389/fpsyt.2025.1596625

**Published:** 2025-07-15

**Authors:** Samantha R. Philip, Erin C. Standen, Jordan Schueler, Sherecce A. Fields, Sean M. Phelan

**Affiliations:** ^1^ Department of Psychological and Brain Sciences, Texas A&M University, College Station, TX, United States; ^2^ Robert D. and Patricia E. Kern Center for the Science of Health Care Delivery, Mayo Clinic, Rochester, MN, United States; ^3^ Department of Pediatrics, Baylor College of Medicine, Houston, TX, United States; ^4^ Division of Health Care Delivery Research, Mayo Clinic, Rochester, MN, United States

**Keywords:** weight bias, weight stigma, weight inclusive care, mental health professional, mental health care, cultural competence, mental health equity, scoping review

## Abstract

**Introduction:**

Weight bias is a pervasive form of prejudice, most deeply and directly harming individuals in larger bodies. Although the mental health field strives to promote the delivery of equitable, culturally sensitive care, the prevalence and nature of weight bias in therapeutic contexts are not well understood. This scoping review examines how weight bias manifests within mental health settings and its impacts on client care and outcomes, exploring the issue from both client and provider lenses.

**Methods:**

A total of 43 studies meeting search criteria were identified from a systematic search process.

**Results:**

Findings indicate that mental health professionals (MHPs) hold negative stereotypes toward larger-bodied individuals. Although MHPs were less likely to report having negative attitudes, they reported a high prevalence of weight bias in their colleagues. Studies using experimental designs demonstrated that providers’ clinical judgment and decision-making were impacted by client body size, generally showing that higher-weight clients were perceived to have lower global functioning, greater pathology, and more negative attributes than lower-weight clients. When the client was described with restrictive eating disorder symptomatology, however, MHPs rated higher-weight clients as less severe and recommended less intensive treatment compared to lower-weight clients. Qualitative studies from client samples revealed experiences of weight stigma during treatment, including MHPs’ expressions of implicit and explicit weight bias, assumptions and misattributions based on the clients’ weight, unsolicited (direct or subtle) weight loss advice, and differential treatment based on size. Experiences of weight bias were harmful to the client’s therapeutic progress and undermined their trust in their provider and the mental health system at large.

**Discussion:**

The body of evidence suggests that weight bias is a serious and significant barrier to the provision of equitable mental health treatment and mental health equity.

## 1 Introduction

Weight bias, defined as negative, prejudicial, or stereotypical beliefs and attitudes directed toward individuals in larger bodies is a well-documented phenomenon impacting the health and well-being of people in larger bodies (1). Prior studies demonstrate that weight bias manifests at structural, institutional, interpersonal, and intrapersonal levels, presenting across life domains (e.g., employment, education, and healthcare) and relationships (e.g., social, familial, and romantic) (2, 3). Experiences of weight stigma are associated with poor mental and physical health outcomes, including increased risk for psychological disorders (e.g., anxiety, depression, substance use disorder, suicidality) (4–6), healthcare avoidance (7), cardiovascular disease markers (4, 5), and a 60% increased risk of death (8). Weight stigma is conjectured to drive health inequities through direct and indirect pathways. The experience of weight stigma has been found to trigger the body’s physiological stress response (9); over time, this chronic stress reaction can increase the allostatic load (10), which is associated with worse health outcomes (11). In medical settings, weight bias is theorized to drive adverse health outcomes through healthcare providers’ biased decision-making and the corrosive effects of provider bias on the patient-provider relationship, leading patients to seek new providers, delay care, or avoid healthcare altogether (12).

When individuals who have experienced stigmatization present to therapy, mental health professionals (MHPs) must understand that clients’ mental health challenges may have been caused or exacerbated by experiences of discrimination (see Meyer and Frost; 13). This recognition represents a facet of cultural competence, which is a core aspect of mental health training programs that is acknowledged in the ethics codes across disciplines (e.g., APA, ACA, NASW, AAMFT). Cultural competence emphasizes self-awareness, knowledge, and skills as a foundation for the provision of high-quality mental health services to individuals of diverse backgrounds (14). Indicators of cultural competence are associated with positive therapeutic outcomes (15). In contrast, perceived microaggressions—defined as “commonplace daily verbal, behavioral, or environmental indignities, whether intentional or unintentional that communicate hostile, derogatory, or negative insults to a target person or group” (16), are negatively associated with therapeutic processes (e.g., therapeutic alliance, perceived cultural humility) and therapeutic outcomes (e.g., improvement in mental health outcomes, satisfaction, and psychological well-being) (17).

Concerningly, the literature indicates that mental health training programs rarely address issues related to weight, including education on weight bias, the complex interaction of factors that influence weight, and how to work with higher-weight clients who struggle with body image or desire to lose weight (18–21). For example, marriage and family therapy trainees, faculty, and clinicians reported that they had not received training on how to effectively work with higher-weight clients, despite treating them in practice (21, 22). Furthermore, a textbook analysis of graduate-level multicultural textbooks revealed that topics of weight stigma and body size as a diversity issue were only addressed in a minority of textbooks, and when addressed, were done so minimally (23). A qualitative study among mental health trainees found that they desire weight bias training to be folded into diversity courses, or to be integrated more broadly throughout training, similar to how identities like race, gender, and sexual orientation are consistently considered (21).

The apparent lack of training on weight bias and weight-related considerations in mental health training programs increases the likelihood that MHPs’ existing biases—shaped by prevailing cultural messages equating weight with health and morality—are left unexamined and unchecked. Indeed, studies indicate that mental health professionals hold weight bias (24–26) and that this bias is perceived by higher-weight individuals (27–29). Drawing from the sizable body of literature in the medical field documenting the detrimental effect of healthcare provider bias on the patient-provider relationship and patient outcomes (7, 30, 31)— and extrapolating from the documented impact of race-based microaggressions on the therapeutic relationship and outcomes (17)— we conjecture that MHPs’ biases may interfere with the therapeutic alliance and treatment progress, potentially reducing individuals’ engagement with mental health services altogether.

The purpose of this scoping review was to examine how weight bias manifests within mental health settings and its impacts on client care, experiences, and outcomes. Specifically, our research questions are: (1) To what extent do MHPs hold bias against higher-weight people? (2) How does provider weight bias influence clinical judgments and decisions? (3) What are the common manifestations of provider weight bias from the client perspective? And (4) What is the impact of perceived provider bias on client experiences? As an emerging body of literature, this scoping review provides a broad overview of the state of the evidence from both client and MHP perspectives. Unless otherwise specified, the terms “mental health professional” and “provider” are used interchangeably to describe psychologists, psychiatrists, therapists, mental health social workers, counselors, and trainees within these fields, and the term “client” is used to describe individuals who received mental health services.

## 2 Methods

To conduct our scoping review, we utilized the methodological framework put forth by Arksey and O’Malley (32). The four stages after identification of our research questions include: identifying potentially relevant studies, study selection, charting the data, and collating, summarizing, and reporting the results.

### 2.1 Literature Search

Key terms were identified to locate studies relevant to the research questions. The following search terms were used: [“weight stigma” OR “weight bias” OR “weight-based microaggression” OR “body size” OR “anti-fat” OR “fat-phobia” OR “fat phobia”] for weight bias, [“therapeutic setting” OR “therapy” OR “mental health treatment” OR “mental health provider” OR “psychologist” OR “psycholog*” OR “social worker” OR “counselor” OR “marriage and family therapist” OR “treatment” OR “milieu” OR “residential” OR “higher levels of care” OR “intensive outpatient” OR “day program” OR “psychological intervention” OR “rapport” OR “clinic”] for mental health settings. The search terms were entered into the databases, combined with the term “and.” To be included, the article needed to be empirical, in English language, and published before December 2024. Review articles and other secondary sources were excluded to ensure the analysis of primary data.

### 2.2 Databases

Five databases were utilized to identify relevant articles: PubMed, APA PsycInfo, ERIC, MEDLINE, and ProQuest eBook Central. PubMed and MEDLINE—both premier resources for biomedical literature—offered access to peer-reviewed research with strong medical relevance (e.g., from medical, psychiatric, and public health journals), providing studies focused on weight stigma in psychological or psychiatric treatment. We utilized APA PsycInfo as a comprehensive resource for peer-reviewed scholarly literature in psychology, providing access to literature focused on behavioral science and mental health, which were of high relevance to our search. ERIC, a database for educational literature, provided empirical literature related to weight bias in educational and training contexts, ensuring that our review included trainee samples. Finally, ProQuest eBook Central provided access to scholarly books, dissertations, and theses, allowing access to essential grey literature rounding out the body of empirical research.

A total of 11,035 articles were found using the above search terms and databases and were imported into Covidence, a tool for conducting reviews and meta-analyses of the literature.

### 2.3 Study selection

**Figure 1:**
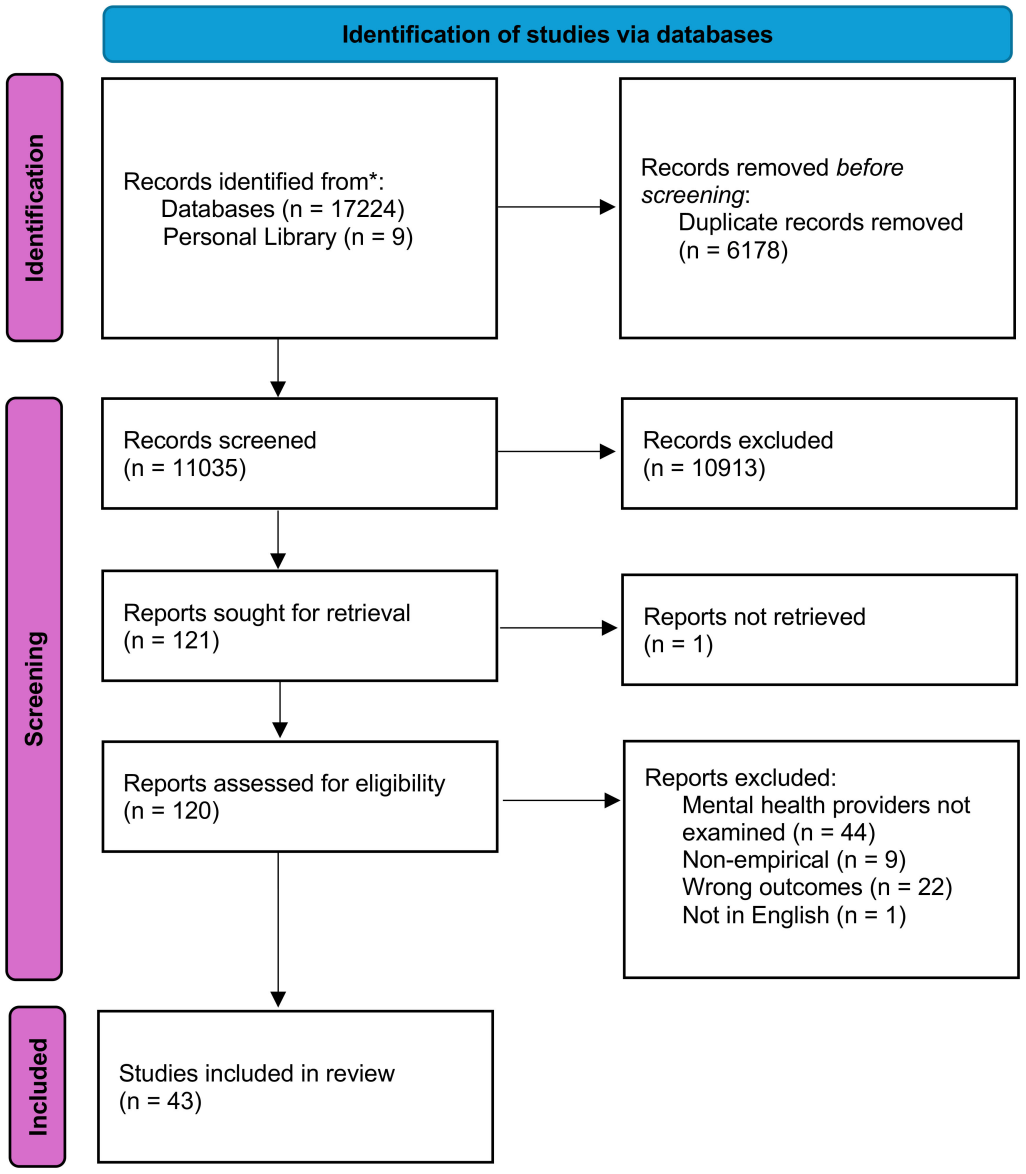
PRISMA flow diagram of identification of articles. Three studies were published as both dissertations and journal articles; each was only counted once.

Five independent reviewers screened titles and abstracts. Each title and abstract were reviewed independently by two reviewers, and conflicts were discussed and resolved by consensus between reviewers with reference to pre-defined criteria. If conflicts persisted, the first author was prearranged to make the final determination, but consensus was reached on all cases. The remaining articles were then subject to a full-text review, by which two independent coders read the full text and determined eligibility. Again, conflicts were resolved through discussion and consensus. Articles were included if they were original studies that examined the presence or impact of weight bias in MHPs, or the experience or impact of weight bias experienced by individuals in mental health settings. Articles were excluded if they did not explicitly measure weight bias in mental health settings or MHPs, or if they were not original research papers (e.g., reviews, perspective papers). Quantitative and qualitative studies were included. This process identified 37 suitable articles. Six additional articles were found as part of the researchers’ library or located in the reference lists of relevant articles. A PRISMA flow diagram (see **Figure 1**) depicts the process and reasons for which studies were included and excluded.

### 2.4 Data charting, collation, and summarization

Data was extracted from each of the identified studies using a Covidence data extraction form modified by the study authors. As our scoping review did not examine intervention studies, we removed all intervention-related details from the template extraction form (e.g., interventions, comparators, exposures, etc.). To capture experimental studies that used a manipulation (e.g., manipulating vignettes by client body size), we inserted a textbox question about manipulation details. Additionally, as our scoping review included studies of qualitative, quantitative, and mixed methodologies—and therefore varied outcomes (e.g., qualitative themes, self-reported measures)—the “Results data” section of the original template was modified to create 15 textbox responses by which study authors could input up to 15 key results from the manuscript.

The charted data included authors, year of publication, study location, study aims, manipulation details (if applicable), study design, study population, sample size, demographic information, outcome measures, and key results. Each step was extracted by two independent study authors to ensure reliability. The data were then thematically organized using deductive followed by inductive qualitative coding scheme. First, a top-down, or deductive, approach was used to create higher-order codes (i.e., by perspective, study design, and context). Next, a bottom-up, or inductive, approach was used, in which all themes and findings were extracted from each study. From there, categories were created by grouping studies with like-themes/findings together.

## 3 Results

### 3.1 Characteristics of Included Studies

The majority of included studies were based in the United States [*n*= 37 (86.0%)], with the remaining studies based in other nations (i.e., Canada, Netherlands, France, Mexico; [*n*= 4 (9.3%)] or international samples [*n*= 2 (4.6%)]. Most studies used observational methodologies [*n*= 18 (31.9%)], followed by experimental [*n*= 11 (25.6%)], qualitative [*n*= 10 (23.3%)], mixed methods [*n*= 3 (7.0%)], and quasi-experimental designs [*n*= 1 (2.3%)]. Of the three mixed methods papers, we only extracted data from the qualitative portion for two (33, 34) as the quantitative data in these studies were not relevant to our research question.

Most studies included mixed-gender samples [*n*= 34 (79.1%)], and the remaining studies included female-identifying participants only [*n*= 2 (4.7%)]. One study (2.3%) used two samples, one with only female participants, and one mixed sample, and six studies (14%) did not report participants’ gender. Approximately two-thirds of studies examined weight bias in samples of mental health professionals [*n*= 29 (67.4%)] and one-third sampled from mental health clients [*n*= 14 (32.5%)]. For study characteristics and key results for MHP and client samples, see **Tables 1** and **2**, respectively.

**Table 1:** Characteristics and key findings of 29 studies examining provider weight bias in MHP samples.

Author, year, and title	Country	Relevant aims	Design	Manipulation details	Population	*N*	Key outcomes measured	Key findings
Mental Health Professional Samples								
Young & Powell (1985) (35). The Effects of Obesity on the Clinical Judgments of Mental Health Professionals	United States	To determine whether a client’s body size affects therapeutic judgments of MHPs	Experimental	Participants received a case description and a photograph of the client that was experimentally manipulated by client body size (“best-weight,” “overweight,” or “obese”), with all other details identical	Interdisciplinary MHPs	120	•**Willingness to Work with Client** (Therapist willingness to work with client) • **Diagnosis, Prognosis, or Treatment Recommendations** (Belief that therapeutic intervention would be useful; belief in a favorable prognosis) • **Perceived Client Attributes** (Perceptions of client level of dysfunction)	•The obese client was assigned more severe symptoms than the non-obese client, and were rated higher on 13/20 symptoms (e.g., impaired judgment, inadequate hygiene, suspiciousness) •Female professionals and younger professionals showed greater evidence of weight bias
Agell & Rothblum (1991) (36). Effects of Clients’ Obesity and Gender on the Therapy Judgments of Psychologists	United States	To investigate whether practicing psychologists who practice therapy assign stereotypical attributions to higher-weight clients	Experimental	Participants received one of two case histories that were experimentally manipulated by client gender (male or female), and body size (“obese” or “non-obese) with all other details identical	Psychologists who were members of APA Division 29	282	• **Perceived Client Attributes** (Person Perception Inventory, including Social Attributes, Appearance, Embarrassment, Softness, and Kindness) • **Diagnosis, Prognosis, or Treatment Recommendations** (Case History Questionnaire, including Problem, Motivation, Self-Concept, Prognosis, Diagnosis)	•The obese client was rated as having more severe problems, being less physically attractive, and being more embarrassed and softer/kinder than the non-obese client
Davis-Coelho et al. (2000) (37). Awareness and Prevention of Bias Against Fat Clients in Psychotherapy	United States	To examine the impact of weight bias on psychologists’ clinical impressions	Experimental	Participants received a case description along with a photograph of the client that was experimentally manipulated by client body size (“average” or “overweight”), with all other details identical	Members and fellows of APA’s Divisions of Clinical Psychology, Counseling Psychology, Psychotherapy, and Psychologists in Independent Practice	200	• **Diagnosis, Prognosis, or Treatment Recommendations** (Psychologists’ recommended treatment modality provision diagnosis; prognosis) • **Perceived Client Attributes** (effort, motivation, overall functioning)	•Fat clients were assigned marginally lower functioning, longer treatment duration, and more severe provisional diagnoses (e.g., eating disorder) than non-fat client •Younger, less experienced, and female providers showed greater evidence of weight bias
Hassel (2001) (38). Client weight as a barrier to non-biased clinical judgment	United States	To explore the impact of client body size on mental health professional’s clinical impressions	Experimental	Participants received a case description that was experimentally manipulated by client body size (“obese” vs. “average”) and gender (male or female), with all other details identical	MHPs currently or recently engaged in clinical work	163	• **Diagnosis, Prognosis, or Treatment Recommendations** (Diagnosis, Global Assessment of Functioning) • **Perceived Client Attributes** (Attitude Scale) • **Explicit Weight Bia**s (Attitudes Toward Adult Obese Patients)	•The obese client was assigned with more pathology (e.g., lower level of functioning), more negative attributes, and more severe diagnoses than the average weight client •Female MHPs showed greater evidence of weight bias in their assignments of global functioning
Adams (2008) (39). Weight Bias Among Counselors-In-Training: A Qualitative Inquiry	United States	To identify and describe the levels of bias that counselors-in-training may have toward higher-weight clients	Experimental	Participants received a case vignette of a client that was experimentally manipulated by client body size (“average” or “overweight”) with all other details identical	Students currently enrolled in graduate-level counseling programs from two universities	56	• Qualitative survey with open-text questions	•Compared to “normal” weight clients, participants used more qualifiers when describing the prognosis, assigned more psychological symptoms, and identified more barriers to effective counseling for the overweight client
Hightower (2014) (40). A Mixed Methods Survey of Fat Bias in Marriage and Family Therapists	United States	To understand the extent that body size and skin color impacted Marriage and Family Therapist’s clinical assessment of a client	Quasi-Experimental	Participants received a case history along with a photograph of the client that was experimentally manipulated by client body size (“average weight” or “overweight”) and skin color (white or dark-skinned) with all other details identical	Licensed Marriage and Family Therapists and Licensed Marriage and Family Therapist-Associates	75	• **Willingness to Work with Client (Therapist willingness to work with client)** • **Diagnosis, Prognosis, or Treatment Recommendations** (General prognosis of client, beliefs about usefulness of therapy) • **Perceived Client Attributes** (Overall assessment of severity) • **Explicit Weight Bias** (Anti-fat Attitude Questionnaire) • Qualitative question probing additional concerns about the client	•Overweight clients were described with unique negative themes (i.e., that were not described for the thin clients), including concerns that the client was suffering from a personality disorder, possible emotional, physical, and/or sexual abuse, and concerns about the client’s weight or potential for having an eating disorder •When controlling for covariates (demographic data), participants rated the average white female client as having greater symptoms of dysfunction than the overweight white female client
Kasardo (2015) (41). Fat Bias in the Field of Psychology: Examining Diversity Counseling Texts and Clinical Judgment Across College Counseling Centers	United States	To examine the impact of client weight labels on clinical impressions of MHPs	Experimental	Participants received an intake report that was experimentally manipulated by client body size (“obese,” “overweight,” “full-figured,” and control)	Interdisciplinary MHPs who worked in college counseling centers	111	• **Willingness to Work with Client** (Interest in working with the client) • **Perceptions of Client Attributes** (Clinical Judgement Questionnaire, Personality Variables) • **Diagnosis, Prognosis, or Treatment Recommendations** (Prognosis, Belief in treatment, Symptoms)	•The obese client was more likely to be recommended weight loss strategies compared to the overweight, full-figured, or control conditions •The obese client was rated as more likely to have eating concerns compared to the full-figured condition, but not compared to the overweight or control clients
Forristal (2018) (42). Fatphobia and Clinical Counseling Decision Making in Counselor Education Students	United States	To examine fatphobia within the context of professional counseling	Experimental	Participants received a case vignette along with a photograph of the client that was experimentally manipulated by client body size (“thin”, “overweight”, or “obese”) with all other details identical	Masters-level counselor education graduate students who had completed their practicum experience	113	• **Diagnosis, Prognosis, or Treatment Recommendations** (Diagnostic Questionnaire) • **Explicit Weight Bias** (Fat Phobia Scale - Short Form) • Weight Bias Internalization Scale	•Participants responses were indicative of explicit weight bias, with especially elevated scores on items describing higher-weight people as: “Slow,” “Weak,” “Insecure,” and having “No willpower” •Compared to the thin or overweight clients, obese clients were more likely to be diagnosed with moderate or severe MDD •Participants reported internalized weight bias on par with those of similar samples
Veillette et al. (2018) (43). What’s Weight Got to Do With It? Mental Health Trainees’ Perceptions of a Client With Anorexia Nervosa Symptoms	United States	To examine the effect of client body mass index (BMI) on diagnostic impressions and perceptions of mental health trainees	Experimental	Participants received a case vignette of a female client presenting for treatment with symptoms of anorexia nervosa; condition was experimentally manipulated by client body size (“underweight,” “normal weight,” or “overweight”) with all other details identical	Graduate-level mental health students	90	• **Willingness to Work with Client** (Treatment Attitudes) • **Diagnosis, Prognosis, or Treatment Recommendation**s (Diagnosis, Number of Treatment Sessions) **• Perceived Client Attribute**s (Endorsement of Weight Stereotypes)	• The overweight client was less likely to be assigned a diagnosis of anorexia nervosa or atypical anorexia nervosa and was recommended fewer treatment sessions than the underweight client
McAshan (2018) (44). The Impact of Client Weight and Ethnicity on Counselor’s Evaluation of Eating Disorder Symptoms: A Vignette Study	United States	To examine how body size and ethnicity influences a counselor’s ability to recognize the presence of, and accurately rate, the severity of eating disorder symptoms	Experimental	Participants received a case vignette of a client describing a young woman with symptoms of anorexia nervosa; condition was experimentally manipulated by client weight (“low” or “high”) and ethnicity (White, Black, Hispanic) with all other details identical	Licensed professional counselors in Texas, California, New Hampshire, New Jersey, and Idaho	306	• **Perceived Client Attributes** (Severity of presenting problem; frequency and severity of client’s symptoms)	•Lower weight clients were more likely to be recommended a medical follow-up, received higher severity ratings, and scored higher on the anorexia subscale than higher weight clients •Participants were more likely to diagnose the lower weight patient with anxiety, and were marginally more likely to diagnose the lower weight client with an eating disorder
Ryland (2020) (45). The Effect Of Anti-Fat Bias On Therapists’ Perceptions Of Client Motivation, Prognosis, Severity Of Mental Illness, And Working Alliance	United States	To evaluate whether MHPs hold anti-fat biases and make different assumptions based on clients’ body size	Experimental	Participants received a case vignette that was experimentally manipulated by client gender (female or male) and body size (normal or obese), with all other details identical	MHPs who were actively seeing clients or had seen clients in the field	213	• **Diagnosis, Prognosis, or Treatment Recommendations** (Perception of potential for a future working alliance; Prognosis Questionnaire) • **Perceived Client Attributes** (Overall assessment of severity; perception of client readiness to change)	•The obese client was rated as less able to develop and achieve therapy goals compared to the average weight client
Silbiger (2024) (46). Mental Health Providers’ Perceptions of Restrictive Eating disorders: Relationship with client body weight	not specified, but researchers are US-based	To investigate how MHPs are influenced by patients’ body weight when evaluating them for symptoms of a restrictive eating disorder	Experimental	Participants received a case vignette for a client that met DSM-5 criteria for either AN or atypical AN; condition was experimentally manipulated by body size (below, within, or above the normal range for her age and height) with all other details identical	Licensed masters’ or doctoral-level MHPs who had been conducting therapy for at least 10 hours per week over the past year	245	• **Diagnosis, Prognosis, or Treatment Recommendations** (Diagnosis free responses, which were then coded; treatment recommendations) • **Perceived Client Attributes** (Assessment of symptoms)	•Low weight clients were more likely to: be labeled with an “eating disorder” or “possible eating disorder” (the two most severe options); be charactered as experiencing “dietary restriction and weight loss;” be recommended “specialized eating disorder treatment;” and be recommended medical follow-up compared to average and high weight clients •Approximately half of participants in the high weight group missed eating disorder symptoms and diagnosis altogether
O’Loughlin (1994) (47). Therapists’ Preferences to Provide Treatment Based on Clients’ Body Size and Gender	United States	To determine whether therapists discriminate against higher weight clients, and more specifically, higher-weight female clients	Observational	N/A	Therapists who either completed a doctoral degree in psychology or were enrolled in advanced levels as a doctoral candidate in a clinical psychology program	128	• **Willingness to Work with Client** (Ranked preference in working with client (obese female, obese male, nonobese female, nonobese male); ranked interest in treating client)	•Female nonobese clients were the most preferred client, while male nonobese clients were the least preferred. The only significant difference emerged between female obese clients and male nonobese clients, with female obese clients preferred to male nonobese clients
McCardle (2008) (48). Weight Bias and Social Work Practice: An Empirical Exploration	United States	To assess weight bias among social work clinicians to determine its potential impact on social work practice	Observational	N/A	Social workers who are members of the National Association of Social Workers and who identified their primary work focus as direct practice	564	• **Explicit Weight Bias** (Attitudes toward obese people, beliefs about weight controllability) • **Diagnosis, Prognosis, or Treatment Behaviors** (Social work practice behaviors with obese clients) • Perceived importance of weight bias in social work practice	•Negative attitudes toward obese people were associated with: believing that obesity is under an individual’s control; lower body mass index; lack of family history of obesity; lack of friends who are obese; lower percentages of obese clients in practice; and older age •Negative attitudes and beliefs were associated with more negative practice behaviors with higher-weight clients •Participants reported fairly high levels of controllability beliefs, especially in their perception of overeating as a primary cause for obesity
Puhl et al. (2014) (49). Obesity Bias in Training: Attitudes, Beliefs, and Observations Among Advanced Trainees in Professional Health Disciplines	United States	To examine weight bias among students training in health disciplines, and to assess the relationship between their weight biases and provision of treatment to patients with obesity, beliefs about the causes of obesity, observations of weight bias in the clinical care setting, and personal characteristics	Observational	N/A	Students enrolled in a post-graduate health discipline (Physician Assistant students, Clinical Psych Interns, or Psychiatric Residents)	107	• **Diagnosis, Prognosis, or Treatment Behaviors** (Expectations of patient treatment compliance/success) • **Explicit Weight Bias** (UMB-FAT; Attitudes toward obese patients; perceived weight bias in health care)	•Students reported high rates of witnessing negative comments/jokes about patients with obesity made by health care providers (65%), by professors or instructors (40%), and by peers (63%), but only 3% of students reported that they themselves believe it is acceptable to make jokes about patients with obesity •Students reported often feeling frustrated with patients with obesity (36%), that patients with obesity lack motivation to make lifestyle changes (33%) and are difficult to deal with (33%). Only 27% of students agreed that treating patients with obesity is professionally rewarding, and 13% indicated that they dislike treating patients with obesity •Participants generally assumed that higher-weight patients would be non-compliant with weight loss recommendations •Participants with more severe personal body shape/weight concerns perceived there to be more weight bias by others in the medical setting
Puhl et al. (2014) (50). Weight Bias among Professionals Treating Eating Disorders: Attitudes about Treatment and Perceived Patient Outcomes	United States	To assess weight bias among professionals who specialize in treating eating disorders and identify to what extent their weight biases are associated with attitudes about treating obese patients.	Observational	N/A	Professionals treating eating disorders, including psychologists, therapists, registered dietitians, social workers, and other	329	• **Explicit Weight Bias** (UMB-Fat, Fat Phobia Scale, Attitudes about Treating Obese Patients) • Perceived Causes of Obesity • Perceptions of Treatment Compliance and Success of Obese Patients	•Providers endorsed negative stereotypes toward higher-weight people, with a sizeable proportion agreeing that: obese individuals have poor self-control (33%), have no willpower (16%), are self-indulgent (15%), are unattractive (24%), are inactive (38%), are insecure (50%), and overeat (55%) •The majority of participants (56%) indicated that they witnessed other professionals in their field making negative comments about obese patients, 42% agreed that other practitioners who treat eating disorders often have negative stereotypes about obese patients, 35% agreed that practitioners feel uncomfortable caring for obese patients, and 29% agreed that their colleagues tend to have negative attitudes toward obese patients •Relatively low percentages of participants (1–17%) expressed negative attitudes about treating obese patients, and high percentages of participants agreed that it is important to treat obese patients with compassion and respect (94%), that treating obese patients is professionally rewarding (72%), and that they feel confident (88%) and professionally prepared (84%) to provide quality care to these patients •Weight bias was inversely associated with BMI and years of professional experience, and positive associated with currently attempting to lose weight
Stokes (2015) (51). Stigma in Clinical Psychology Trainees: Bias Towards Eating Disorders on the Basis of Weight Variance and the Mediating Influence of Personal Psychological Traits	United States	To explore the presence and impact of weight stigma and eating disorder stigma in graduate-level psychology trainees	Observational	N/A	Clinical PsyD students	117	• **Explicit Weight Bias** (UMB-FAT)	•A small proportion (i.e., 3 to 21%) of trainees endorsed explicit weight bias •Participants with BMIs in the “normal weight” category reported higher levels of general weight stigma than those in the “obese” category
Soto et al. (2014) (52). Beliefs, Attitudes and Phobias Among Mexican Medical and Psychology Students Towards People with Obesity	Mexico	To evaluate the beliefs and attitudes that Mexican medical and psychology students have towards obese people	Observational	N/A	Students from the first and last year of the School of Medical and Psychology at the Autonomous University of Baja California (UABC)	528 (278 psychology students & 250 medical students)	• **Explicit Weight Bias** (Beliefs about Obese Persons Scale, Attitudes Toward Obese Persons Scale, Fat Phobia Scale)	•Compared with medical students, psychology students had better knowledge about the causes of obesity, and less negative attitudes and beliefs towards people with obesity •Psychology students’ weight bias was on par with that of the general population •Over 40% of the sample of psychology students endorsed negative adjectives of obese people, including “Likes food,” “Overeats,” “Slow,” “Poor self-control,” “Inactive,” “Shapeless,” and “Low self-esteem”
Pratt et al. (2016) (26). Marriage and Family Therapy Trainees’ Reports of Explicit Weight Bias	United States	To explore levels of explicit weight bias and identifying demographic factors associated with bias among MFT students	Observational	N/A	MFT students currently enrolled in COAMFTE programs	162	• **Explicit Weight Bias** (Beliefs about Obese Persons Scale, Attitudes Toward Obese Persons Scale, Anti-fat Attitudes Questionnaire)	•Evidence of explicit weight bias was found •Explicit weight bias was higher among students who were white, masters (vs. doctoral) students, and who identified as overweight
Darling & Atav (2019) (53). Attitudes Toward Obese People: A Comparative Study of Nursing, Education, and Social Work Students	United States	To assess the attitudes of graduate and understand students toward obese population, and compare the attitudes of nursing students to those in other professional fields	Observational	N/A	Undergraduate and graduate nursing students and graduate education and social work students at a northeastern university	440 (56 social work students)	• **Explicit Weight Bias** (Attitudes Toward Obese Persons Scale, Beliefs about Obese Persons Scale)	•Social work students had significantly more positive attitudes toward obese people than nursing students, and significantly lower controllability beliefs than nursing and education students
Lee (2019) (54). Graduate Training in Body Image Complexity: Evolving Competence to Meet Emerging Research	United States, Canada	To explore students’ potential biases and confidence in addressing body image in practice	Observational	N/A	Training directors: Current training director with minimum of 1 year in current program Doctoral students: Current student with minimum of 1 year in current program and minimum of 1 completed semester of clinical experience at the doctoral level in an APA- or CPA-accredited program	21 training directors; 114 doctoral students	• **Explicit Weight Bias** (Modified Attitudes about Treating Obese Patients Scale) • Experiences of Body Image Training and Education	• Most students reported that their programs did not effectively encourage their self-reflection of personal size as a cultural identity (59%) or their exploration of their personal biases and assumptions of larger individuals (62%) • Students and training directors reported that other health providers in their field have stereotypes toward larger-bodied clients/patients (student endorsement 49%; director endorsement 65%) and that they have heard/witnessed other professionals make negative comments about larger-bodied clients/patients (student endorsement 42%; director endorsement 47%). Overall, students and training directors reported low explicit weight bias • Most participants (76%) noted that bodies are either discussed “rarely” or not discussed at all within their programs, and approximately half of student participants (49%) reported feeling incompetent working with body image in session
Christensen (2021) (55). Factors Related to Weight-Bias Among Counselors	United States	To examine factors that contribute to weight-bias among licensed counselors	Observational	N/A	Counselors who earned a master’s degree in counseling	587	• **Explicit Weight Bias** (Fat Phobia Scale - Short Form)	• Explicit weight bias was positive associated with male identity, and inversely associated with weight bias education and multicultural competence
Brochu (2023) (56). Testing the Effectiveness of a Weight Bias Educational Intervention Among Clinical Psychology Trainees	United States	To test the efficacy of a weight bias seminar on reducing weight controllability beliefs, anti-fat attitudes, and attitudes toward fat clients	Experimental*	N/A	Clinical psychology trainees (i.e., clinical psychology graduate students, predoctoral interns, and postdoctoral fellows)	56 baseline observations	• **Explicit Weight Bias** (Attitudes Toward Fat Clients Scale, Anti-fat Attitudes Questionnaire- Dislike and Willpower subscales)	• Evidence of moderate levels of weight-controllability beliefs, anti-fat dislike, and negative attitudes toward fat clients
Franko (2023) (57). The Correlates of Explicit Weight Bias among Mental Health Providers in Training	United States	Examine the prevalence and correlates of explicit weight bias among MHPs who are in training	Observational	N/A	Trainees currently enrolled in master’s and doctoral programs in mental healthcare fields	287	• **Explicit Weight Bias** (Beliefs about Obese Persons Scale, Attitudes Toward Obese Persons Scale, Anti-fat Attitudes Questionnaire)	• Trainees reported greater negative attitudes and beliefs toward higher-weight people than a community sample of UK adults • White racial identity was associated with higher levels of explicit weight bias, while non-binary/other gender identity and more years in graduate school were associated with lower levels of explicit weight bias
van der Voorn et al. (2023) (58). Weight-Biased Attitudes about Pediatric Patients with Obesity in Dutch Healthcare Professionals from Seven Different Professions	Netherlands	To study the prevalence and interdisciplinary differences of weight-biased attitudes of Dutch HCPs who treat children and adolescents with obesity, including MHPs (only 7% of sample, but separated out for subgroup analyses)	Observational	N/A	Dutch healthcare professionals who treat children/adolescents with obesity	555 (40 MHPs)	• **Explicit Weight Bias** (Attitudes toward treating patients with obesity, including negative attitudes towards patients w obesity; perceived frustrations in treating these patients; perceived confidence and preparedness to treat patients w obesity; perceived weight bias by colleagues)	• Compared to other disciplines (e.g., pediatricians, GPs), dieticians and mental health professionals reported some of the lowest negative weight-based attitudes and lowest frustrations with higher-weight patients • MHPs reported similar levels of perceived bias from colleagues in their field as other disciplines
Philip et al. (2024) (25). Comparisons of Explicit Weight Bias Across Common Clinical Specialties of US Resident Physicians	United States	To examine how explicit weight bias varies across individuals in common residency specialties	Observational	N/A	Second year residents from 49 allopathic medical schools	3267	• **Explicit Weight Bias** (Anti-Fat Dislike, Anti-Fat Blame, Attitudes Toward Obese Patients)	• Psychiatry residents were grouped with residency specialties (e.g., family medicine, pediatrics) reporting the lowest levels of explicit weight bias compared to other medical residency specialties (e.g., anesthesiology, orthopedic surgery)
Sohier et al. (2024) (59). Bias Related to Overweight and Obesity among French Psychiatrists: Results of a National Survey	France	To assess factors that may influence weight-related bias among psychiatrists, to explore the relevance of visual assessment of body mass index, and to determine how they this feature is integrated into their practice	Observational	N/A	Senior psychiatrists and residents in psychiatry	271	• **Explicit Weight Bias** (Beliefs about Obese Persons Scale, Fat Phobia Scale)	• Psychiatrists exhibited anti-fat bias, with higher levels found in residents (vs. senior physicians) • 76% of the psychiatrists reported that they inquired about their patient’s weight more than never, while 66.4% reported that they do not systematically assess for the presence of overweight or obesity in their patients. 31.7% of the participants reported that it was somewhat challenging to inquire about their patients’ weight •87.5% of respondents indicated a concern for prescription adjustments based on the patient’s weight
Aza (2009) (60). What’s the Skinny on Fat Women in Psychotherapy: Mental Health Clinicians’ Countertransference with Women of Size	United States	The researcher sought to explore MHPs’ experiences of countertransference with women of size	Qualitative	N/A	MHPs’ from various mental health backgrounds in Georgia who have worked with at least one fat female client	12	• Interviews (45–60 minutes) conducted face-to-face	• Most participants indicated some form of weight bias toward higher-weight women • MHPs commonly reported affective responses such as devaluation, fear, shame, and confusion when working with higher-weight women, which could manifest as microaggressions • Many MHPs described questions about how to “help” their clients with their weight, citing health concerns
Hedden (2023) (61). Novice Counselors’ Weight and Body Image Beliefs: An Exploratory Study	United States	To understand novice counselors’ attitudes and beliefs about weight and body image	Mixed Methods	N/A	Novice practicing counselors who graduated from CACREP-accredited clinical mental health programs within the last three years and are practicing in southern US	24	•**Explicit Weight Bias** (Assortment of Q set statements) •Qualitative survey with open-text responses	• Participants were sorted into four factors: Body Positivists (n=7), Body Liberators (n=4), Body Choosers (n=5), and Body Changers (n=2) • Body Positivists aligned with opinions that promote body acceptance and celebrating all bodies, simultaneously endorsed beliefs that higher body weight is associated with worse health; Body Liberators aligned with a social positive of fat activism, taking a firm position against diets and diet culture, and supporting counselors’ roles in providing fat-affirming care. None of these participants were trying to lose weight.; Body Choosers expressed opinions that assigned individual responsibility for higher weight, and framed obesity as a chronic disease that must be addressed by healthcare providers, overtly rejecting the idea of fat liberation and fat-affirming care. All participants on this factor were either trying to lose or maintain weight; Body Changers (n=2) endorsed beliefs that obesity is a chronic disease and that healthcare providers should address weight, but did not endorse stereotypes about fat people are assign morality to overeating. This group also highlighted racial differences in body standards between Black and White women. All participants on this factor were Black women trying to lose weight.

*Only observational data extracted.

**Table 2:** Characteristics of 14 studies examining provider weight bias in client samples.

Author and year	Country	Relevant aims	Design	Population	*N*	Key outcomes measured	Key findings
Patient Samples							
Downes (2001) (64). What Do Fat Women Want? An Exploratory Investigation of the Influences of Psychotherapy on the Process by Which Fat Women Work Toward Acceptance of Their Size and Weight	United States	To present and describe fat women’s experiences in therapy and current reflections upon those experiences	Qualitative	Fat women (BMI at least 34) at least 30 years of age who are engaged in the process of working toward accepting rather than changing her size and weight, and who have been or currently are clients in psychotherapy	10	• Two interviews (1.5–2 hours) conducted (interview setting not reported)	•Participants reported that they had difficulty raising weight-related struggles in therapy, expressing fear that they would lose the trust they had built with the therapist, or that they would be made to feel further shame about their bodies/their attempts to accept their size •Although none of the participants went to therapy with weight loss as a goal, some participants reported that their therapists suggested that they lose weight. Those who experienced a therapist’s suggestion that they lose weight– or the suggestion they were “in denial” if they spoke in terms of accepting their bodies– reported that these suggestions adversely impacted the therapeutic relationship •Participants uniformly expressed a preference for a therapist who is aware of how genetics influence body size, understand the about issues facing fat women, and is comfortable with their body
Ciepcielinski (2016) (65). Client Perceptions of Weight Stigma among Eating Disorder Professionals	United States	To explore client perceptions of weight stigma among eating disorder professionals and assess clients’ perception of its impact on treatment and quality of care	Qualitative	Individuals who perceived weight stigma among eating disorder professionals who they either received or were currently receiving eating disorder treatment for BED or related symptoms	10	• 1–2 interviews (45–50 minutes) conducted via phone or videoconference	•Participants perceived that the needs of clients with binge eating disorder were less highly prioritized compared to those with anorexia or bulimia, and that ED professionals lacked adequate knowledge regarding weight stigma and binge eating •Experiences of weight stigma in ED treatment were harmful to the client (e.g., triggering emotional distress and ED symptoms), the patient-provider relationship, and the client’s eating disorder recovery
Raves et al. (2016) (33). Bariatric Surgery Patients’ Perceptions of Weight-Related Stigma in Healthcare Settings Impair Post-Surgery Dietary Adherence	United States	To explore provider and patient perspectives on adherence and stigma in healthcare settings	Mixed Methods*	Eligible participants had enrolled in a pre-surgical preparatory program prior to bariatric surgery or in the 24-month post-surgery	35	• Three interviews (45–120 minutes; initial, 4–8 months later, then 4–8 months after that) conducted (interview setting not reported) • Observations of participants conducted over multiple years in bariatric clinic practice from which they were recruited	•Participants described MHPs’ rigidity around diets and food rules following surgery, and lack of understanding for participants’ lived experiences and responsibilities that could make adherence difficult •Participants desired mental health treatment following their surgeries, but felt that MHPs were often ill-equipped for their case
Akoury et al. (2019) (27). Fat Women’s Experiences in Therapy: “You Can’t See Beyond … Unless I Share It with You	United States	To examine patient accounts of weight-based stigma and discrimination in therapy and their advice for therapists who work with fat women	Qualitative	Women with BMI in the “obese” range who had at least one therapy session within the last 6 months	15	• Semi-structured, face-to-face interviews (45–60 minutes)	•Participants reported that providers made assumptions about their mental health based on their body size, and appeared less interested in and engaged with them based on their size •Negative feelings associated with weight made participants less forthcoming, more evasive, and more avoidant in session or of sessions (e.g., missed sessions due to weight-related concerns) Participants reported instances of furniture and spaces in the therapy office that were not size-inclusive •Participants advised therapists to recognize fat women as a whole person, and to allow the client to bring in their concerns about weight
Abel (2020) (66). “Let’s Talk About Your Weight”: How Fatphobia Manifests in Therapy	Canada	To explore the experiences of people who have discussed their weight and body size in therapy	Qualitative	Participants either had been or were currently in therapy, and were members of Facebook Groups: Fat Awesome and Queer (FAQ), Fat Babes Society, Fat Friends, Fat Activists, Fat Fitness and Well-Being, and Curvy Palz	16	• Semi-structured, face-to-face narrative interviews (1.5–2 hours)	•Participants reported that their therapists believed or insisted that their weight was central to their psychological challenges, while ignoring the impact of other key factors •Participants described therapists’ expressions of overt and implicit weight bias •Participants avoided body-related discussions, believing that therapists lacked the skills to explore fatphobia in ways that would benefit them and fearing their judgment •Participants reported experiences with therapy spaces that were not set up to accommodate all body sizes, including tight spaces and furniture that was too small or not sturdy •Participants reported that their therapists’ disclosure of their own weight-related struggles were inappropriate, uncomfortable, and detrimental to the participant’s therapeutic progress •Participants recommended that therapists: navigate conversations around size by challenging fatphobia and avoiding linking mental health with body size; become educated on anti-fatness as a form of oppression; allow the client to first raise weight-related topics; include neutral body-related intake questions or body positive signifiers in the office; and avoid making diet and exercise recommendations/bringing up weight as a problem
Moore (2022) (67). Exploring Higher Weight Women’s Experiences of Provider Weight Stigma	United States	To explore the phenomena of weight stigma among higher weight women in mental health treatment who also engage in restrictive eating behaviors	Qualitative	Adult women who wear a pant/dress size 14 or above, who struggled with emotional and behavioral restrictive eating behaviors, and who have sought mental health treatment in the past 5 years. Participants who were formally diagnosed with binge eating disorder were excluded	8	• Semi-structured interviews (60–90 minutes) conducted via videoconference	•Participants described experiences of weight-based microaggressions, including providers suggesting or pushing dietary restriction/weight loss •Participants experienced MHPs pathologizing fatness and suggesting personality responsibility for their body size •Participants experienced harm to their self-image and relationship with self and body as a result of provider bias, damaging their journey to body acceptance •Provider bias harmed the therapeutic relationship, making it feel unsafe, and increased reluctance and fear of seeking future help
Goehner (2023) (68). Finding Body Appreciation Through the Weight-Neutral Framework	United States	To understand how weight-neutral treatments promote body appreciation among higher-weight women	Qualitative	Weight neutral sample: women between 25–45 years old who wear a pant size of 16 or higher, and who had at least six sessions with a weight-neutral provider. Weight-focused sample: Women who underwent bariatric surgery.	9 (6 in weight-neutral group, 3 in weight-focused group)	• Interviews (40–90 minutes) conducted via videoconference	•Participants reported that MHPs did not adequately address body image or lacked knowledge about weight-neutral approaches or people in larger bodies generally •Participants described weight bias statements or assumptions by MHPs that made them feel shame and anxiety about their health care •Some participants in the weight-focused group reported that their psychological treatment following bariatric surgery was not holistic, either ignoring important details about the participant’s food intake (e.g., that the participant was not eating enough) or focusing too much on food-related teaching
Harrop et al. (2023) (28). “You Don’t Look Anorexic”: Atypical Anorexia Patient Experiences of Weight Stigma in Medical Care	United States	To investigate the lived experiences of individuals with atypical anorexia nervosa	Qualitative	Adult women and non-binary persons assigned female at birth who experienced atypical anorexia nervosa.	38	• Semi-structured interviews (1.5–4 hours) conducted (interview setting not reported)	•Participants faced weight stigma in higher levels of ED care (i.e., intensive outpatient, partial hospitalization, and residential treatment) including differential treatment on the basis of size, witnessing providers ignore fatphobic comments made by patients, and receiving encouragement from MHPs to continue disorder behaviors while they were in recovery (e.g., recommending diets and weight loss) •Participants reported that providers minimized their EDs and cited examples of misdiagnoses and missed symptoms, often due to assumptions that participants were “overeating” or binge eating due to their size •Participants believed that chronic undertreatment lengthened their illness trajectories
Gilbert (2024) (34). Atypical Anorexia Nervosa: Examining the Impact of Weight Stigma on Weight Bias Internalization and Eating Disorder Symptoms	United States	To understand how weight stigmatizing experiences influenced current eating disorder symptoms and experiences of eating disorder treatment for adults with atypical anorexia nervosa	Mixed Methods*	Adults who have received treatment for atypical AN and encountered weight stigma.	30	• Qualitative survey	•90% of participants identified weight stigmatizing encounters with at least one of their ED providers, including providers making inaccurate size-based assumptions that led to patient neglect; dismissing their health concerns; failing to conduct appropriate assessments and diagnose; failing to provide appropriate treatment; describing high body weight as a negative quality; prescribing more restrictive meal plans; and praising weight loss •All but one participant reported these encounters negatively impacted their treatment and recovery, including reduced trust in providers, heightened ED symptoms, and future mistrust in ED healthcare and ambivalence/hopelessness about recovery
Sonnenblick et al. (2024) (69). Behavioral Weight Loss Treatment for Adults with Binge-Eating Disorder: A Qualitative Analysis of Patients’ Perspectives and Experiences	United States	To inform clinical practice for adults with BED and overweight/obesity by collecting and synthesizing patients’ perspectives on whether, how, and for whom BWL should be offered	Qualitative	Briefly: Adults with BED with a BMI between 27 and 45	45	• Client Eating Disorder Pathology (Eating Disorder Examination Questionnaire) • Qualitative interviews (duration not reported) conducted via videoconference	•All participants reported that they did not feel stigmatized for their weight by the behavioral weight loss treatment they received or by their therapists •Many participants believed that behavioral weigh loss was possible without stigma, especially if the treatment had a non-judgmental group environment, focused on health aspects of weight loss, and is voluntary •Some participants thought that behavioral weight loss is inherently stigmatizing, but that the societal emphasis on thinness (rather than the treatment itself) is at fault
Talbert (2024) (70). An Examination of the Lived Experiences of those who have Received or Attempted to Receive Treatment and/or Recovery from Atypical Anorexia in a Higher Weight Body	Not specified, but researchers are US-based	To explore the lived experiences of individuals who received or attempted to receive treatment and/or recovery from atypical anorexia nervosa at a higher body weight	Qualitative	Assigned female at birth, 18+, BMI 25+, received or attempted to receive treatment for AAN	8	• Semi-structured interviews (60 minutes) conducted via videoconference	•Participants unanimously described treatment as harmful and/or inadequate, with a detrimental focus on weight restoration, restrictive meal plans, and even weight loss •Most participants reported experiences of their BMI/size impacting their treatment quality and progress, including feeling doubted in their ED, receiving differential treatment from thinner counterparts, and being prescribed medication to lose weight •Many participants described a fear of seeking treatment for eating disorders due to past experiences of weight discrimination or bias during treatment and from medical providers, interfering with their recovery •Participants reported that their care was harmed by providers’ lack of education on atypical anorexia
Puhl & Brownell (2006) (29). Confronting and Coping with Weight Stigma: An Investigation of Overweight and Obese Adults	United States	To examine experiences of weight stigmatization, sources of stigma, coping strategies, psychological functioning, and eating behaviors in higher-weight adults	Observational	Adults with membership in a national non-profit, non-commercial weight loss support group organization with active chapters across the country	Sample 1 = 2449 (female only); Sample 2 = 222 (matched sample)	• **Client Experiences or Sources of Weight Stigma** (Interpersonal sources of stigma) • Coping Responses to Weight Stigma	•In the first sample, 21% of participants reported experiences of weight stigma from MHPs on at least one occasion, and 13% reported multiple occasions •In the second sample, 13% of women and 12% of men reported experiences of stigma from MHPs more than once and multiple times
Puhl et al. (2021) (63). International Comparisons of Weight Stigma: Addressing a Void in the Field	Australia, Canada, France, Germany, United Kingdom, United States	To assess experiences and interpersonal sources of weight stigma in adults	Observational	Members of weight watchers international in Australia, Canada, France, Germany, the UK, and the US	13,996	• **Client Experiences or Sources of Weight Stigma** (History of experienced weight stigma, Interpersonal sources of weight stigma, Weight Stigma Time of Life Questionnaire)	•Mental health professionals were identified as source of stigma by 11.8% of participants
Chen & Gonzales (2022) (62). Understanding Weight Stigma in Eating Disorder Treatment: Development and Initial Validation of a Treatment-Based Stigma Scale	Not specified, but researchers are US-based	To psychometrically validate the Scale for Treatment-based Experiences of Weight Stigma (STEWS) for patient-centered assessment of weight-stigmatizing experiences in eating disorder treatment	Observational	Former eating disorder patients with a body mass index greater than 25	142	• **Client Experiences or Sources of Weight Stigma** (Scale of Treatment-Based Experiences of Weight Stigma) • **Client Eating Disorder Pathology** (Eating Disorder Examination Questionnaire- Short)	•Treatment-based experiences of weight stigma (measured by STEWS) was significantly and positively associated with eating disorder symptomatology •The STEWS score was found to contribute to variance in eating disorder symptomatology above and beyond the variance explained by BMI, weight stigma in everyday life, and weight bias •46.4% of the sample agreed that their providers recommended dieting even when they did not come in to discuss weight loss, and 40.0% agreed that their providers supported disordered eating behaviors or attitudes in service of weight loss •28.2% those who struggled with restrictive behavior agreed that their providers overlooked or disregarded treating these symptoms, and 26.0% of those who struggled with compensatory or purging behaviors agreed that their providers overlooked these symptoms

*Only qualitative data extracted.

### 3.2 Findings from MHP Samples

#### 3.2.1 Experimental studies

A total of eleven experimental (35–46) and one quasi-experimental (40) studies evaluated the impact of client body size on clinical decision-making in MHPs. Of the 12 total studies, nine described a client with general mental health challenges, and three described a client presenting with eating pathology. These categories are summarized separately due to the unique manifestations of weight bias in an eating disorder context.

##### 3.2.1.1 Impact of body size on perceptions of general psychopathology

Seven of nine studies (35, 36, 38–41, 45) measured the MHP’s perception of the client’s psychological severity and examined differences by weight condition. Four studies found that higher-weight clients were assigned greater psychological severity than lower-weight clients (35, 36, 38, 39). One study found the opposite trend, with the lower-weight condition being assigned greater dysfunction than the higher-weight condition (40). Two studies found no difference in perceived symptom severity across weight condition (41, 45).

Six of nine studies (36–38, 40–42) assessed MHP’s provisional diagnosis and/or treatment goals for the client. Four studies found differences by clients’ described body size, including higher-weight clients being more likely to be diagnosed with an adjustment disorder when lower-weight clients were more likely to be diagnosed with relational problems (38); an eating disorder when average-weight clients were more likely to be diagnosed with an adjustment disorder (37); and moderate or severe MDD (42). Further, respondents were more likely to indicate “increasing sexual satisfaction” and weight loss as treatment goals for clients described as “fat” or “obese” (37, 41). Two studies did not find differences by client body size (36, 40).

Five of nine studies (35, 36, 38, 40, 41) measured symptom attributions that MHPs made about the described client. Four of the studies demonstrated that higher-weight clients were rated more negatively than lower-weight clients (38), such as being rated higher on symptoms including agitation, emotional behavior, impaired judgment, and inadequate hygiene (35), being rated less attractive and more embarrassed (36), or being described as suffering from a personality disorder or possible emotional, physical, and/or sexual abuse (40).

Four of nine studies (35, 40, 41, 45) measured the MHP’s interest in working with the client. Across studies, no significant differences were found based on client body size. One observational study also found no differences in provider preference or interest in working with clients based on body size (47). One study found significant differences on a subscale measuring the MHP’s belief in the client’s ability to achieve their therapy goals, with lower-weight clients being ranked more favorably than higher-weight clients (45). Finally, seven of nine (35–37, 39–41, 45) studies measured the predicted prognosis for the client. Six studies found no difference by body size (35, 36, 39–41, 45). One study found that the higher-weight client was expected to have a longer course of treatment (37).

###### 3.2.1.1.1 Interactions with provider attributes

Four studies examined interaction effects by MHP attributes (35, 37, 38, 41). Female providers demonstrated a higher degree of weight bias than male providers (35, 37, 38) in three of four studies. Age also emerged as a significant moderator, with younger MHPs tending to demonstrate more biased responses (35, 37) in two studies, with the opposite pattern found in one study (41).

##### 3.2.1.2 Impact of body size on perceptions of eating pathology

All three studies (43, 44, 46) described a client with symptoms consistent with a restrictive eating disorder. Each of the studies examined how MHPs assigned diagnoses and symptoms to the client based on the client’s body size. The studies consistently reflected that MHPs were less likely to consider restrictive eating disorder pathology for higher-weight clients. For example, one study found that clients described as “overweight” were less likely to receive a diagnosis of anorexia nervosa or atypical anorexia nervosa than those described as “underweight,” (43), and another found that MHPs were more likely to label the lower-weight client with an eating disorder or possible eating disorder (46). Silbiger’s (2024) study also demonstrated that 53.2% of MHPs completely missed the presence of eating disorder symptoms in the client. Across studies, providers assigned higher symptoms of anorexia to the lower-weight client compared to the higher-weight client conditions (43, 44, 46).

All three studies assessed providers’ judgments surrounding treatment planning and/or referrals. Across studies, higher-weight clients were perceived as needing less care, with providers recommending fewer treatment sessions (43), being less likely to schedule a medical follow-up (44, 46), and being less likely to be recommended specialized eating disorder treatment. Relatedly, McAshan’s (2018) study found that the lower-weight client’s eating disorder was perceived as significantly more severe than the higher-weight client’s (44).

#### 3.2.2 Observational studies

Sixteen studies used observational methodologies to examine the presence of provider weight bias (25, 26, 42, 47–59), 13 of which measured explicit weight bias in MHPs (25, 26, 42, 48–54, 56–59). Two of the aforementioned studies also examined MHPs’ feelings of competence for working with larger-bodied clients (49, 54). One observational study (47) examined MHPs’ ranked preference for working with clients based on body size; these findings were described above given the stronger conceptual fit.

##### 3.2.2.1 Prevalence of explicit weight bias

Evidence of weight-stigmatizing beliefs and attitudes in MHPs emerged across the 13 studies (25, 26, 42, 48–54, 56–59) measuring anti-fat attitudes. Multiple studies found evidence of MHPs endorsing negative stereotypes about higher-weight people (42, 49, 50, 52, 56, 59), and some found evidence of MHPs’ endorsement of negative attitudes toward higher-weight people or clients (56, 57, 59), though providers endorsed negative attitudes at low rates relative to their endorsement of negative stereotypes (51, 54, 58). In studies that compared MHPs to other medical professionals (e.g., pediatricians, GPs, nursing students), MHPs consistently reported lower levels of weight bias (25, 53, 58). Interestingly, one study found that—despite lower self-reported weight bias—MHPs reported similar levels of perceived weight bias among colleagues as in other disciplines (58). Two other studies reflected similar patterns; although MHPs endorsed relatively low levels of weight bias in themselves, they indicated a high degree of weight bias exhibited among colleagues in their field (49, 54).

###### 3.2.2.1.1 Demographic diLerences in weight bias

Nine of the 13 above studies (26, 48–50, 52, 55–57, 59) examined differences in provider bias by gender, age, weight and related experiences, race/ethnicity, and training or experience level. Of the five studies (52, 55–57, 59) that examined how gender influenced weight bias, four studies found no differences in anti-fat attitudes or beliefs between men and women (52, 56, 57, 59), but one study found that MHPs who identified as nonbinary had lower controllability beliefs than those who identified as men or women (57). One study found that being male was associated with higher levels of weight bias (55). Two studies examined the impact of age, with one study finding that younger providers reported more tolerance for higher-weight clients (48), while another study found no differences by age (52). Three studies examined the role of racial and ethnic differences (26, 56, 57), with two studies finding that white MHPs held higher levels of weight bias than non-white MHPs (26, 57) and one study finding no differences by race (56).

Five studies examined the role of training and years of experience on provider biases, with consistent evidence that more years of experience was associated with less weight bias (26, 49, 50, 57, 59). Furthermore, one study reflected that receiving training on weight bias was negatively associated with weight bias (55). Five studies (48, 50, 52, 56, 57) examined the role of the MHPs’ weight and weight-related experiences; three studies found that weight bias was not influenced by MHPs’ BMI/perceived weight (52, 56, 57) or body concerns (57). In contrast, two studies found that weight bias was inversely related to higher BMI (48, 50), as well as a family history of “obesity”, having more higher-weight friends, and having a higher percentage of clients in larger bodies (48). One study found that MHPs with higher eating disorder symptoms observed more weight bias among their peers and providers in healthcare settings (50). One study found that MHPs who were actively attempting to lose weight endorsed more negative attitudes about treating higher-weight clients (50).

##### 3.2.2.2 MHP preparedness to work with higher-weight clients

Two studies (49, 54) examined MHPs’ comfort working with higher-weight clients. In Lee et al.’s (2020) study that sampled clinical and counseling psychology doctoral students of APA-accredited programs, 58.2% of trainees indicated that they would feel comfortable broaching body image in a session, but 49.1% indicated that they would feel incompetent working with body image in a session. In contrast, a study sampling from eating disorder providers found that they largely felt confident (88%) and professionally prepared (84%) to provide quality care to higher-weight clients (49).

#### 3.2.3 Qualitative and mixed-methods studies

Two studies that shed light on providers’ weight bias utilized qualitative methods (60, 61). Hedden’s (2024) study used a Q methodology to understand early counselors’ attitudes and beliefs about weight and size, while Aza’s (2009) study involved interviews with MHPs to understand their internal reactions to higher-weight clients. Both studies suggested that most providers feel compelled to help their higher-weight clients lose weight, citing concerns about health at higher body weights. Hedden’s (2024) study also revealed that a subgroup of MHPs overtly rejected the notion of fat liberation and fat-affirming care, while another subgroup disagreed with the idea of providers not assisting with weight loss and simply holding space for clients (61).

Aza’s (2009) study—focused on MHPs reactions to female clients in larger bodies—found that most providers endorsed weight bias toward higher-weight women, and experienced intense affective responses in their presence, including devaluation, fear, shame, and confusion. Some providers in the study described microaggressions they committed toward higher-weight clients, including providing higher-weight female clients with unsolicited weight loss advice (microinsult) and subtly dismissing a client’s feelings when she described a recent experience of weight discrimination (microinvalidation) (60).

Both studies also found evidence of a small subgroup of providers with weight-inclusive mindsets and practices. In Hedden’s (2024) study, the authors found that a small subgroup of providers (4 of 24) took a firm position in opposition to diets and diet culture and believed in fat-affirming care. In Aza’s (2009) study, 3 of 12 providers used size-inclusive language and described the value of normalizing and celebrating diverse body shapes and sizes.

### 3.3 Findings from client samples

#### 3.3.1 Observational studies

Three observational studies examined clients’ reports of weight stigma from MHPs (29, 62, 63). Puhl and Brownell’s (2006) study included two samples. In the larger female-only sample (*N*=2,440), 21% of participants reported that they had experienced weight stigma from MHPs on at least one occasion, and 13% reported that they had multiple experiences of weight stigma from MHPs. In the second, mixed-gender sample where men and women were matched for age and BMI (*N=*222), 13% of women and 12% of men reported weight stigma from mental health providers on multiple occasions (29). In a subsequent study using an international sample (i.e., Australia, Canada, France, Germany, United Kingdom, United States) of adults enrolled in Weight Watchers International, 11.8% of participants reported experiencing weight stigma from a MHP at least once. No national differences were found (63).

The third study sampled participants with a history of an eating disorder and with a body mass index greater than 25 (62). Nearly half of the sample (46.4%) endorsed that their MHPs recommended dieting even when they did not come in to discuss weight loss, and 40% of participants agreed that their providers were in support of disordered eating behaviors and attitudes in service of weight loss. Of those who struggled with restrictive behaviors and compensatory/purging behaviors, 28.2% and 26.0%, respectively, reported that their providers overlooked or disregarded those symptoms (68).

#### 3.3.2 Qualitative Studies

A total of 11 qualitative studies (27, 28, 33, 34, 64–70) examined experiences of weight stigma in mental health settings from the client perspective. Of the 11, six were from participants in general mental health settings (27, 33, 64, 66–68), and five (28, 34, 65, 69, 70) were from participants in eating disorder settings. Due to the unique manifestations of weight bias in eating disorder settings, these subgroups are reported separately.

##### 3.3.2.1 Common manifestations and impacts of weight stigma in general outpatient treatment settings

Provider weight bias was described by participants as most commonly manifesting through MHPs’ subtle and overt communication around exercise, body size, and weight loss (66, 67), suggestions of personal responsibility for body size (67), nonverbal cues (e.g., appearing less interested and engaged with them; 27), and MHPs’ overemphasis on clients’ weight, leading them to mis-conceptualize clients’ challenges (27, 66–68). Four studies found that participants reported that providers made unsolicited weight loss recommendations (27, 64, 66, 67) and further doubled down on their weight loss agenda despite participants’ desire to work on accepting their bodies (66, 67). Three studies found that MHPs engaged in self-disclosure around their weight and weight-related behaviors (27, 66, 67), with one study demonstrating that almost half of the participants reported their providers self-disclosing along these lines (27). Participants from two studies reported that these self-disclosures were inappropriate and detrimental (66), making the space feel less safe for clients healing from disordered eating (67). In the two identified studies involving individuals that underwent bariatric surgery, participants reported MHPs’ over-focus on food-related teaching and rules that were not aligned with clients’ holistic needs for therapy (33, 68).

Three qualitative studies (64, 65, 68) documented the impact of perceived provider weight bias on the client and/or the therapeutic relationship. Each study demonstrated serious consequences of experiences of mental health professionals’ weight bias. Participants described how provider weight bias undermined the therapeutic relationship, making the therapeutic relationship feel unsafe, reducing trust, and increasing participants’ reluctance to seek help from future MHPs (64, 67). Additionally, provider weight bias stunted clients’ therapeutic progress, with participants describing how provider bias damaged their self-image and relationships with themselves, heightened shame and anxiety, and compelled them to question their journey of self- and body-acceptance (67, 68). In turn, participants reported feeling more disconnected from their bodies and poorer relationships with food and exercise (67).

Four studies (27, 64, 69, 70) uncovered themes related to clients’ willingness to discuss their weight with their MHPs. All four studies found that participants were reluctant to bring up their weight in therapy and/or that their weight (and associated shame or self-consciousness) made them more evasive and avoidant in therapy sessions (27, 64, 66, 68). Two studies found that participants reported explicitly avoiding or fearing having discussions about their bodies with their provider for fear of judgment or a poor reaction from the provider that could undermine trust and safety (64, 66). One study found that participants believed that MHPs lacked the necessary skills to help them in this realm (66).

Four studies demonstrated participants’ sentiments that MHPs lack sufficient training in body image and weight-neutral approaches (33, 66–68). In turn, participants felt that their body image struggles were not adequately addressed, or that they were made to feel that they were at fault for not meeting beauty standards, as opposed to being encouraged to reflect on body-based systems of oppression. In two studies, participants described frustration about their need to educate their providers on weight stigma (66, 67). Three studies found that some participants preferred working with MHPs who were also fat, as this shared identity could promote a sense of trust through joint lived experiences and understanding (64, 66, 68).

Four studies (27, 65, 67, 70) inquired into participants’ recommendations for MHPs to better service higher-weight clients in therapy. Three studies included a theme that highlighted participants’ wish for providers to be aligned with fat-positive or Health at Every Size^®^ principles, including rejecting mainstream narratives around body size and taking a holistic, person-centered approach that recognizes the person as more than their weight (27, 64, 66). Relatedly, participants in all studies described a need for providers to become educated on weight-related matters, including the common issues faced by higher-weight people (27, 64, 65), the biological determinants of size (64), and anti-fatness as a form of oppression (66).

In terms of concrete ideas for creating more inclusive practices, participants suggested providers include body-related questions in their intakes (66)—but ask about eating in the same way that they might ask a smaller-bodied client (64)—, include body-positive and inclusive signifiers in their office space (64, 66), and ensure that their office furniture accommodates larger bodies (64, 67). Participants in one study strongly recommended against MHPs making diet and exercise recommendations (66), while some participants in another study expressed a desire for therapists to help them with their weight-loss goals (27). Generally, participants agreed that MHPs should allow clients to bring up the topic of their weight and that they should not bring up weight as a problem (27, 66). Participants in three studies emphasized the importance of providers not making assumptions about a client based on their body size–especially assuming causal links between their size and their mental health issues (27, 64, 66).

##### 3.3.2.2 Common manifestations and impacts of weight stigma in eating disorder treatment settings

Of the five studies (28, 34, 65, 69, 70) examining client experiences of weight stigma in eating disorder treatment, three studies utilized samples who had sought or received treatment for atypical anorexia nervosa (28, 34, 70), and two studies utilized samples who had received treatment for binge eating disorder (65, 69).

The three studies focused on individuals in larger bodies with atypical anorexia found evidence of widespread encounters of provider weight bias in this setting (28, 34, 70), with 90% of participants in one study (*N*=30) reporting that they had encountered weight stigma from an eating disorder provider (34). While one study documented participants’ direct observation of providers describing high body weight as a negative quality (34), another study found that treatment providers did not address fatphobic comments made by other clients (28). In contrast, one study among recipients of a behavioral weight loss treatment for binge eating disorder found that participants largely denied feeling stigmatized by the behavioral weight loss treatment that they received, or by their providers (69).

One of the most common manifestations in eating disorder settings—emerging across four of the five studies—was the experience of differential treatment from treatment providers because of their size (28, 34, 70) or diagnosis (e.g., binge eating disorder vs. anorexia nervosa/bulimia nervosa) (65). Participants described a sense that their illnesses were taken less seriously, and their needs were prioritized below, their peers in smaller bodies (28, 34, 65, 70). For example, participants in one study reported that their providers viewed high weight as indicating that one is not “actually sick” with an eating disorder (34). This experience was apparent even in higher levels of eating disorder care (i.e., intensive outpatient, partial hospitalization, and residential treatment), where participants reported that providers were less likely to believe the symptoms of higher-weight clients compared to lower-weight clients (28, 34). The experiences of dismissal and disbelief were even more pronounced for individuals with multiple oppressed identities (28).

Providers’ weight bias reduced the quality of care provided to higher-weight clients, skewing their clinical judgments and the treatment offered to them. Two studies focusing on participants with a history of restrictive eating disorders found that MHPs misdiagnosed their illness or missed restrictive symptoms, instead assuming that the participant was binge eating or “overeating” due to their body size (28, 34). Both studies found evidence of provider negligence, by which they failed to conduct thorough assessments for accurate diagnosis and appropriate treatment (28, 34). These biased assumptions led to suboptimal, and even harmful, treatment decisions. Despite sharing the same symptoms as their smaller-bodied peers, participants reported receiving different interventions and care recommendations (e.g., more restrictive meal plans), and not receiving the necessary care for their eating disorder (e.g., group therapy for food restriction) (28, 34, 70). Participants commonly reported providers *actively encouraging eating disorder behaviors* while they were in recovery from a restrictive eating disorder, including recommending or praising weight loss and restrictive eating (28, 34, 70).

Participants from four of the five studies (28, 34, 65, 70) uniformly reported negative impacts of MHP weight bias on the therapeutic relationship and on the participant’s recovery. Provider weight bias diminished participants’ trust in treatment providers, harming relationships within and outside of the treatment team- including undermining general trust in eating disorder healthcare (34, 65, 70). These experiences interfered with client recovery in several ways, including heightened self-doubt, negative self-stigma, internal anguish (65), and greater difficulty developing a healthy relationship with food, eating, and their bodies and accepting their bodies’ dietary needs (34). Ultimately, provider stigma resulted in increased eating disorder symptoms and restriction (34, 65), which participants reported using as a means of self-protection from provider weight stigma (34). Participants in three studies described how provider stigma lengthened their illness trajectories and/or posed additional barriers to recovery, such as fear of seeking future treatment (28, 34, 70).

## 4 Discussion

Weight stigma is a known risk factor for reduced mental health and wellbeing of higher-weight individuals. The extent to which weight bias may appear in the therapeutic context—potentially posing further harm to client and therapeutic processes—was previously not well-defined. Synthesizing insights on this topic from both client and MHP perspectives, qualitative and quantitative investigations, and published journal articles and dissertations, this scoping review sought to comprehensively map this phenomenon and to answer the following questions: (1) To what extent do MHPs hold bias against higher-weight people? (2) How does provider weight bias influence clinical judgments and decisions? (3) What are the common manifestations of provider weight bias from the client perspective? And (4) What is the impact of perceived provider bias on client experiences? The findings of this scoping review highlight the exacerbating process by which higher-weight individuals may face further psychological harm when seeking mental health services due to provider weight bias.

We found conclusive evidence that MHPs hold weight bias toward larger-bodied individuals and clients, converging across observational, qualitative, experimental, and mixed methodologies. The findings suggested that MHPs may be reticent to disclose their negative attitudes toward higher-weight individuals, but they openly endorse stereotypical beliefs about higher-weight people (e.g., that they are insecure, unattractive, or have poor self-control) (42, 48, 54, 56, 57), and report high perceptions of bias among their professional colleagues (49, 54). MHPs reported having strong affective reactions to women of size, described examples of weight-based microaggressions toward clients, and demonstrated weight-centric beliefs (e.g., that weight is under one’s control) (60, 61).

Numerous experimental studies sought to examine how weight bias influences MHPs’ clinical judgments and decisions. Though findings varied across studies, general trends indicated that, compared to smaller-bodied clients with otherwise identical presentation, providers perceived higher-weight clients in general mental health settings as having greater dysfunction, more severe diagnoses, and more psychological challenges and symptoms (35–39). Most studies did not find differences in MHPs’ self-reported interest in working with the client or the clients’ predicted prognosis by clients’ body size. When examining this question in the context of eating disorders, MHPs consistently perceived larger-bodied clients’ restrictive symptomatology as less severe, less diagnosable, and in need of less medical attention compared to smaller-bodied clients (43, 44, 46).

Qualitative studies from client samples illustrate the manifestation and consistently negative impact of perceived MHP weight bias and weight-related discussions on client experiences and outcomes. The results suggested that many clients suffered from their MHPs’ reinforcement of the thin ideal, by which MHPs encouraged clients to lose weight without their asking, self-disclosed about their personal pursuits of thinness via diets and exercise, and made clients feel as though their bodies were “wrong” and not worthy of acceptance (64, 67). Clients described providers dismissing their key mental health concerns to focus instead on their weight, with some MHPs insisting that their weight was central to their psychological challenges or that their body was to blame for their mental health concerns or the trauma they had suffered (27, 66, 67). Other clients described experiences of providers’ equally hurtful subtle weight bias, by which they observed MHPs appearing less interested and engaged with higher-weight clients within a group therapy context (27). Experiences of MHP weight stigma induced shame, anxiety, and self-doubt, increased internalized weight stigma, reduced body trust among clients, and caused clients to question their journey of body/fat acceptance (67, 68). Furthermore, experiences of provider stigma made the therapeutic relationship feel unsafe, undermining trust in the provider and the mental health field at large (64, 67), and making it more difficult to bring up their body-related challenges in therapy.

The negative impacts of provider bias were equally, if not more, destructive in eating disorder treatment settings. As in general outpatient settings, clients reported that providers encouraged them to lose weight and engage in restrictive eating behaviors while they were actively in recovery from restrictive eating disorders (28, 34, 62, 70). Presumably based on assumptions that higher-weight clients must “overeat,” provider bias commonly led providers to overlook or doubt restrictive symptoms in higher-weight clients, fail to conduct appropriate assessments, and misdiagnose clients (28, 34, 70). In turn, clients reported that they did not receive the level or type of care that they needed. Participants consistently reported sentiments of differential treatment on the basis of body size, in which they observed their lower-weight peers being prioritized and taken more seriously (28, 34, 65). Experiences of weight stigma in eating disorder treatment settings resulted in a breach of trust between the client and their treatment providers, diminished quality of care, heightened eating disorder symptoms and psychological distress, and a lasting negative impact on eating disorder recovery by undermining clients’ trust in eating disorder healthcare generally (28, 34, 70).

The results from the scoping review also illuminated how weight bias manifests on structural levels within the therapeutic context and confers harm on the client. One example of this structural stigma emerged in clients’ reports of the therapeutic space being unaccommodating to bodies on the higher end of the weight spectrum, including tight spaces and small or insubstantial furniture (27, 66). When therapeutic settings are not set up to comfortably service all clients, it can signal to clients that they are unwelcome and pose an immediate barrier to the therapeutic work. Another example includes findings from MHP samples that reveal a lack of graduate training and sense of discomfort supporting clients with body image issues. One study demonstrated that over 75% of participants reported that bodies (e.g., weight, size, ability state) are “rarely” or “not at all” discussed within their programs (54). The lack of training on weight- and size-related issues is likely reflective of an implicit, structural-level bias, impacting the content that graduate programs deem important or unimportant. Studies from the client perspective make clear how the omission of training harms clients’ experiences in therapy; providers’ lack of knowledge and education on pertinent topics (e.g., body-based oppression, lived experiences of higher weight people) and therapeutic techniques (e.g., weight-neutral approaches) can force clients into the educator role with their therapists (66–68). This need to educate was described as frustrating and burdensome by clients (67), and often led to clients evading discussions of weight with their MHP (27, 64, 66, 68).

Through not a primary aim, several manuscripts in this scoping review sought feedback from clients about how MHPs could cultivate more inclusive and effective practices for higher-weight individuals. Participants consistently described a need for providers to become more knowledgeable about lived experiences of higher-weight individuals, including anti-fatness as form of oppression, the politics of fatness, the biological determinants of size, and how size impacts one’s experience (27, 64, 66). Frequently, participants described a desire to work with fat-affirming providers who were aligned with weight-inclusive approaches, rejecting the mainstream narratives around body size and pressures for thinness (64, 66). Participants emphasized the need for providers to avoid making assumptions pertaining to how their body size relates to their history or presenting problems (27, 64, 66) and to focus on the client as a whole person rather than assuming that weight is a central issue (27). To create an environment in which clients feel more safe to talk about their experiences in their bodies, some clients recommended that therapists include size inclusive signifiers in their office, inquire about eating behaviors in the same way they might ask a smaller-bodied person, provide size-friendly spaces and seating, and avoid bringing up weight as a problem, recommending diets or exercise, or disclosing about their personal pursuit of weight loss (64, 66, 67).

## 5 Limitations and future directions

The findings of this scoping review should be considered within the context of their limitations. First, we acknowledge methodological limitations inherent in the study design and execution. While our use of search tools was deliberate and broad, it is possible that some manuscripts were not indexed by any of the search tools used, and therefore not included in this review. Additionally, the use of only published literature may skew our findings toward more significant results (i.e., reflecting publication bias (71)), though our inclusion of theses and dissertations attenuates this concern.

Another methodological limitation was the omission of search terms related to psychiatrists and psychiatric behaviors. Despite not including such terms, several studies including psychiatrist samples were returned in our search and included in this review. Still, this omission limits our ability to draw conclusions about this subgroup, and particularly the impact of clients’ weight status on physicians’ prescription decisions. A review of available experimental research in this population would provide important insights into this clinical decision-making process that is highly susceptible to weight bias.

Some limitations of this review result from limitations of the available evidence. Compared to studies examining provider bias by sampling MHPs, far fewer studies examined provider weight bias and its impacts from the client perspective, and most of these studies used qualitative methodologies. The qualitative findings provided nuanced, in-depth insights into their experiences, but have some limitations to their generalizability due to the smaller sample sizes, and they do not allow us to quantify or draw causal conclusions regarding the impact of provider bias on client outcomes and treatment decisions. Additional observational studies are needed to quantify the effect of MHPs’ perceived weight bias on the therapeutic relationship and client outcomes (e.g., psychological well-being, future mental healthcare utilization). Future research should also employ experimental methodology to examine the effect of provider weight bias on relevant client outcomes.

Additionally, our search returned few studies examining MHP bias in settings other than general outpatient and eating disorder treatment settings. The field’s understanding of this issue will be advanced by expanding the examination of weight bias to encompass a broad range of clinical settings (e.g., intensive outpatient/partial hospitalization, inpatient) and modalities (e.g., individual therapy, group therapy, couples therapy) and clinical populations (e.g., mood, anxiety, serious mental illness).

Finally, given the focus of this scoping review, we did not examine how MHPs might transition to becoming more weight-inclusive practitioners. Very few interventions to our knowledge have examined the impacts of weight bias reduction interventions in mental health trainees (56, 72), and few qualitative studies have examined the personal and professional work of MHPs specializing in body image concerns (73, 74). Generally, such providers endorsed a weight-inclusive approach, acknowledging body diversity, understanding sizeism as a form of oppression, and rejecting mainstream diet culture and weight-centric beliefs about weight and health. These studies also called attention to the need to examine one’s own relationship with their bodies to best serve their clients and for more formal training within graduate school and counseling organizations. Investigating and understanding the processes by which MHPs unlearn harmful weight-based beliefs and embody weight-inclusive, harm reduction practices represent an essential area of future research.

## 6 Conclusions

The results of this scoping review suggest that weight bias is a serious issue in mental health settings, in need of attention and remediation. While future research is needed, it is evident that MHPs hold stigmatizing views toward higher-weight clients and that their clinical judgments and decisions are impacted by this bias. Given the negative mental health impact of weight stigma, this is especially concerning; clients may encounter the same form of stigma from MHPs that originally contributed to the development or exacerbation of their mental health challenges. The impacts of provider bias—suggested by the findings of this review— are that clients feel less safe with their providers, experience heightened mental health symptoms, are reluctant to share their true thoughts and feelings about their bodies, and are discouraged from seeking future treatment. Increased efforts in education, training, and research are needed to promote size-inclusive beliefs and practices in mental health trainees and professionals, such that therapy can be a safe and affirming space for people of all sizes.

## Data Availability

The original contributions presented in the study are included in the article/supplementary material. Further inquiries can be directed to the corresponding author/s.
